# Co-creating a 24-hour movement behavior tool together with 9–12-year-old children using mixed-methods: MyDailyMoves

**DOI:** 10.1186/s12966-020-00965-0

**Published:** 2020-05-14

**Authors:** Lisan M. Hidding, Mai J. M. Chinapaw, Laura S. Belmon, Teatske M. Altenburg

**Affiliations:** 1 Amsterdam UMC, Vrije Universiteit Amsterdam, Department of Public and Occupational Health, Amsterdam Public Health research institute, van der Boechorststraat 7, 1081BT Amsterdam, the Netherlands; 2 Department of Epidemiology & Health Promotion, Section Youth, Municipal Health Service Amsterdam, Nieuwe Achtergracht 100, 1018WT Amsterdam, the Netherlands

**Keywords:** Photovoice, Concept mapping, Focus groups, Co-creating, Online self-report tool, Physical activity, Sedentary behavior, Sleep

## Abstract

**Background:**

All 24-h movement behaviors, i.e. physical activity, sedentary behavior and sleep, are important for optimal health in children. Currently, no tools exist that include all 24-h behaviors and have been proven to be both reliable and valid. Potential reasons for the inadequate validity and reliability of existing questionnaires are the lack of focus on the content validity and lack of involvement of children in the development. Therefore, the aim of this study was to co-create a 24-h movement behavior tool together with 9–12-year-old children.

**Methods:**

Concept mapping and photovoice meetings were held to identify children’s physical activity behaviors. During concept mapping meetings with four groups of children (*n* = 40), children generated an extensive list of physical activities they engaged in, sorted the activities in categories and rated the frequency and perceived intensity of these activities. Using photovoice, three groups of children (*n* = 24) photographed their physical activities during one weekday and one weekend day, named the photographs, and placed them on a timeline. Furthermore, researchers obtained information on relevant items regarding sleep and sedentary behavior by screening existing questionnaires. Thereafter, we developed the first version of MyDailyMoves. Subsequently, we examined the content validity of the tool together with three groups of children (*n* = 22) and one group of researchers (*n* = 7) using focus group meetings.

**Results:**

MyDailyMoves has a timeline format, onto which children add the activities they performed the previous day. Based on the concept mapping and photovoice studies, eight physical activity categories were included: playing inside, playing outside, sports, hobbies, chores, personal care, transport, and others. Sleep questions and two more sedentary categories (schoolwork and screen time) were added to MyDailyMoves to define and complete the timeline. The content validity study showed that all items in the tool were relevant. However, children mentioned that the activity category ‘eating’ was missing and the understandability of how to use the tool should be improved by adding an explanatory video. Both suggestions were adopted in the second version.

**Conclusion:**

Including the children’s perceptions throughout the tool development process resulted in a comprehensive and practical tool which is easy for children to use.

## Background

Recently, the importance of all 24-h movement behaviors, including physical activity, sedentary behavior and sleep, for optimal health in children has emerged [1]. A large body of evidence exists for the relationship between childhood physical activity and various health benefits [2]. Evidence for an adverse relationship between sedentary behavior and children’s health is inconclusive [3, 4]. Nevertheless, the growing public health concern regarding the health effects of excessive sedentary behavior has led to recommendations to limit sedentary (screen) time [5–7]. In addition, healthy sleep in children is associated with various health benefits [8]. Yet, few children meet the physical activity recommendations [9, 10], the majority of children spend a large amount of their time sedentary [11, 12], and sleep duration seems to decline [13, 14].

In order to monitor all 24-h movement behaviors in larger child-populations and to assess the effectiveness of behavioral interventions, adequate, affordable, and convenient measures of these behaviors are needed. Accelerometry is considered to be valid and reliable for assessing physical activity and valid in assessing sleep duration in children [15, 16]. Furthermore, inclinometry has shown to be a valid measure of sedentary behavior in children [17]. However, the data of these measures lacks information on the context of the behavior. Furthermore, subjective decisions are needed to convert the data into time estimates of physical activity, sedentary behavior and sleep, e.g. definition of non-wear time, number of valid days required, and choice of cut points to define the intensity of activity. On the other hand, self-report is regarded as a convenient and affordable way to assess physical activity, sedentary behavior and sleep including contextual information, i.e. the type and location of children’s activities [18].

To date, none of the available physical activity and sedentary behavior questionnaires for children have acceptable validity and reliability [19–21], and to our knowledge, no self-report measure including all 24-h movement behaviors exists with both acceptable validity and reliability. Questionnaires are not without limitations, e.g. social desirability and recall bias are major issues [18], which may partly explain the lack of valid and reliable self-report measures. Another possible explanation may be the lack of focus on content validity, which is defined as ‘the degree to which the content of the measurement instrument is an adequate reflection of the construct to be measured’ [22]. Content validity is one of the most important measurement properties of self-report measurement tools [23]. Nevertheless, a description of the development of a questionnaire is often lacking, and the content validity is often not examined or is minimally described [20, 21]. Moreover, children themselves are rarely involved in studies examining the relevance, comprehensibility, and comprehensiveness of questionnaire items [20, 21]. Consequently, there may be a gap between the recalled activities in existing questionnaires and activities that children mostly engage in.

As children can be valuable experts of their own behavior [24], children’s perceptions are essential when it comes to developing valid and reliable measurement instruments measuring their behavior. Therefore, we aimed to develop a measurement tool (MyDailyMoves) together with 9–12-year-old children. Our initial aim was to co-create a tool measuring children’s physical activity and the context of their activities, which explains our primary focus on physical activities in the conducted studies. However, as the importance of all 24-h movement behaviors for optimal health emerged during the study [1], we reformulated our aim to include all children’s activities, including both physical and sedentary activities as well as sleep, within MyDailyMoves.

Therefore, our final aim was to co-create a 24-h movement behavior tool for primary school children together with 9–12-year old children, by: 1) examining children’s perception of physical activity using the concept-mapping method; 2) examining children’s physical activity behavior and the context of their behavior using the photovoice method; 3) screening the literature on relevant questionnaire items regarding sleep and sedentary behavior, and 4) assessing the content validity of the newly-developed MyDailyMoves together with children and researchers in the fields of child public health, measurement tool development, physical activity, sedentary behavior and sleep.

## Methods

### General procedures

**Fig. 1:**
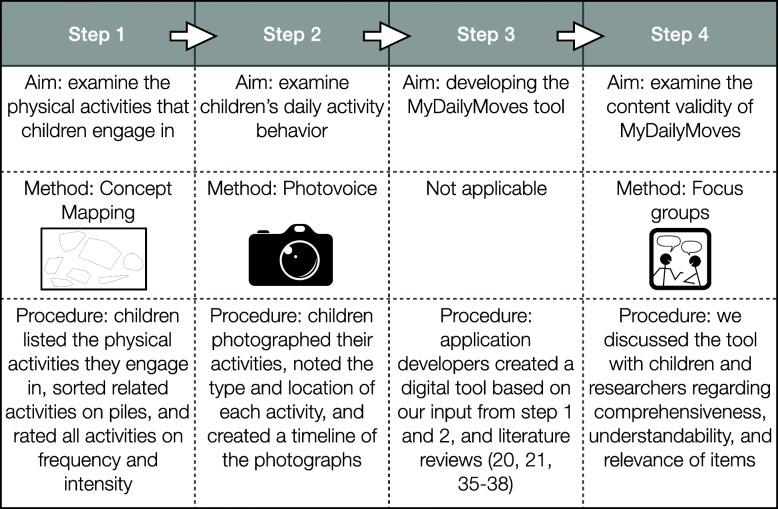
Overview of steps undertaken in the co-creation of MyDailyMoves. Pictures by Pixabay

For the development and the content validity assessment of the MyDailyMoves measurement tool, the consensus-based standards for the selection of health measurement instruments (COSMIN) content validity guideline was followed [23]. In short, we included the key populations’ perception of physical activity using qualitative methods (concept mapping and photovoice), and data were collected until saturation was reached. Furthermore, both children and researchers were asked about the relevance of items, comprehensiveness, and comprehensibility in the content validity assessment. For this study, four steps were followed, and three different methods were applied (see Fig. 1). First, the types, intensity, and frequency of physical activity that children engage in were explored using concept mapping (step 1; data collection between April and June 2016). Concept mapping is a method in which group perceptions are examined using a qualitative data collection and a quantitative data analysis [25, 26]. Second, children’s physical activities, and their locations were examined using photovoice (step 2; data collection between September 2016 and February 2017). Photovoice is a method in which children use photography to express their ideas and share their opinions, e.g. about their physical activity practices, by talking about their photographs [27–30]. Next, a measurement tool (MyDailyMoves) was developed (step 3). In this step, existing questionnaires regarding sleep and sedentary behavior were screened to include relevant items regarding these behaviors in the tool. Lastly, the content validity of MyDailyMoves, which included comprehensiveness, understandability and relevance of items/questions, was examined in focus group discussions (step 4; data collection between September and October 2018) with both children and researchers in the fields of child public health, measurement tool development, physical activity, sedentary behavior and sleep. Based on these results, the tool was adapted. The detailed procedures of step 1, 2 and 4 are described below. The development of MyDailyMoves (step 3) is described in theResults section. Study size for the concept mapping and photovoice study was determined by data saturation; study size for the content validity study was based on the COSMIN guideline [23].

### Participants

For the concept mapping and photovoice studies, participants in the 6th, 7th or 8th grade (9–12 years old) were recruited via primary schools. This age range was chosen as our previous experiences show that children from the age of 9 are cognitively able to fill in self-report tools and to participate in the participatory methods used. Schools were selected through purposive sampling in order to include children attending primary schools located in urban and rural areas and with different socioeconomic backgrounds. The socio-economic status (SES) of the primary schools was divided in tertiles, i.e. low, medium, and high SES, and obtained using the school zip-code and the status-scores document from the Dutch Social and Cultural Planning Agency [31]. Primary schools were approached by telephone, e-mail, or via the personal network of the researchers. A total of seven primary schools were willing to participate: four schools participated in the concept mapping study and three in the photovoice study (response rates 25 and 100%, respectively). If a school agreed to participate, information letters were sent to the parents and children of the participating grade. For the content validity study, children (9–12 years old) were recruited via two locations: an after-school care facility and one primary school. A maximum of nine children were allowed per focus group. The after-school care facility locations and primary school were selected through convenience sampling, i.e. via the researchers’ professional and personal networks. Furthermore, the researchers that participated in the content validity study were also recruited via the personal network of the researchers of this study. Information letters were sent to all participants of the study, i.e. the researchers participating in the content validity study, the children, and the children’s parents. Of the children who consented for participation, eight, nine and ten were randomly selected for the photovoice, content validity, and concept mapping study, respectively. As a reward for participation, children received a small gift related to physical activity, e.g. a frisbee or simple pedometer. Primary schools were offered an information meeting about the study results.

The VU University Medical Ethical Committee approved the study protocols (nrs. A2016.328; 2016.209; 2018.314;). No identifying participant information was collected for the purpose of this study, and written informed consent was signed by one parent and the participating child. Furthermore, the researchers participating in the content validity study also signed written informed consent.

### Step 1 - Concept mapping

Detailed concept mapping procedures are previously published [32, 33]. In short, two separate meetings with four groups of children (*n* = 10 per group) were organized (one group per school) at the children’s school. The aim of the first meeting was to obtain information on the physical activities that children engage in. As an introduction, children answered the following warm-up question: ‘What do you enjoy doing or what are your hobbies?’ The children had to assess whether the activities they wrote down were active, inactive, or could be both. Next, children brainstormed about the physical activities that they engage in by means of a main question, which was formulated as a question and a sentence:

‘*What physically active activities do you do during the day?’*

*‘The physically active activities that I do during the day are…’*

Children generated responses to the main question, resulting in a list of unique activities of children from each school. In the second session children were instructed to sort all the generated activities into piles of related activities and subsequently name the piles. Thereafter, children rated the activities on intensity (ranging from: not tired at all to so tired, I can’t go anymore) and frequency (ranging from: never to every day) using 5-point Likert scales.

Concept mapping software ‘Ariadne’ was used to analyze children’s sorting and rating data [34]. Concept maps were created by multidimensional scaling and hierarchical cluster analysis. For each school, a separate concept map was created, including eight clusters by default. Mean ratings for frequency and intensity were calculated for each cluster.

Two researchers independently interpreted the concept maps. First, they determined the final number of clusters and named each cluster by interpreting the underlying activities of each and adopting the names that the children used in the sorting task. At two schools the concept maps were also interpreted by the children by interpreting the underlying ideas within each cluster and naming the final clusters. As some of the clusters represented more than one main topic, i.e. activity category, we refer to activity categories instead of clusters for the remainder of the manuscript.

### Step 2 – Photovoice

Photovoice meetings with three groups of children were conducted at their schools (*n* = 6–8 per group; one group per school). Three to four weekly meetings per group were held; children decided whether a fourth meeting was necessary. All meetings were recorded with a voice recorder, and each child received a camera that he/she could keep for the entire research period. The first introduction meeting started with a photography lesson given by a photographer and a researcher. Children learned about six photography topics, i.e. frame, subject of the photograph, position, color, background, and moment. Furthermore, children learned about photography ethics and rules, e.g. safety while making photographs and that other children/people should not be recognizable in their photographs. Next, all children received their cameras and went on a photo expedition in their schoolyard with the photographer and researcher. They practiced with photographing their active activities, could work together with other children, and could ask questions to the researcher and the photographer. At the end of the meeting the children received a homework assignment: ‘photograph your physically-active activities for two weekdays and one weekend day, from the moment you wake up until you go to bed again’. Each child received a notebook to clarify the activities they photographed in case a photograph ended up blurry. During the second, third and optional fourth meeting children analyzed the qualitative data themselves by discussing the activities they photographed and placing all photographs on a timeline. First, the children were provided with a print of their photographs, which they divided in a weekday- and a weekend-day-pile. Next, the children coded their photographs by adding notes, i.e. they wrote down what activity they were doing and where the activity took place. Thereafter, all children were instructed to create a timeline together (separate for a weekday and a weekend day) using a piece of wallpaper and a marker and to place their photographs on the timeline. If children remembered an activity they had not photographed, they wrote that activity on a sticky note and placed it on the timeline. The timeline started at the time the children woke up and ended at the time they went to bed. Additionally, the children wrote down other time periods, e.g. the start of school and school breaks, which helped them with accurately placing their photographs. As a preparation for the next session the children were asked whether there were activities missing on the timeline that they could photograph the next week. Data saturation was achieved when all children concluded that there were no missing activities; due to data saturation, the next session was cancelled.

Two independent researchers identified the activities and their locations by interpreting the photographs and their descriptions. Thereafter, activities and locations were categorized using the activity categories identified in the concept mapping study.

### Step 3 – Development of MyDailyMoves

MyDailyMoves is an online measurement tool in the form of a website. Its development is based on the following studies: 1) a systematic review on the measurement properties of physical activity questionnaires for children [20]; 2) the photovoice study (described in this paper); 3) the concept mapping study (described in this paper); 4) a systematic review on the measurement properties of sedentary behavior questionnaires for children [21]; and 5) literature screening on relevant studies on sleep behavior, sleep questionnaires [35–37], and experiences gained by pilot-testing a sleep diary as part of an ongoing research project [Belmon et al., unpublished]. The first study was used to gain insight in the useful aspects of existing questionnaires measuring physical activity in children, with the aspects being included in MyDailyMoves. The second and third study, i.e. the concept mapping and photovoice study (described in this paper), were used to create activity and location categories to be included in MyDailyMoves. Main categories were used instead of recording all individual activities to minimize the burden for children; also, we were more interested in the intensity of the activities than the specific activity within an activity domain. The fourth study was used to obtain information regarding sedentary categories that should be included in MyDailyMoves. Lastly, the sleep questions were developed by first defining the relevant sleep domains, i.e. sleep duration, efficiency, timing, quality and daytime sleepiness [38]. We chose to measure all sleep domains as this provides an overview of children’s sleep health, which is more than merely the duration of sleep. Per sleep domain, relevant questions were selected based on the literature for sleep duration (i.e. time in bed) [35], sleep efficiency (i.e. sleep onset latency and night wakings) [35, 37], sleep timing (i.e. the placement of sleep within 24 h day), sleep quality (i.e. satisfaction with own sleep) [35], and daytime sleepiness [36, 37]. To limit the burden on children, we limited the number of sleep-related questions to a maximum of six with at least one question per domain. Completing (the first version of) MyDailyMoves took the children between 15 to 30 min.

### Step 4 - content validity focus groups

Three focus groups with children (*n* = 5–9 per group) and one focus group with seven researchers in the fields of child public health, measurement tool development, physical activity, sedentary behavior and sleep (from Amsterdam UMC: Department of Public and Occupational Health and Department of Epidemiology; Municipality of Amsterdam: Department of Epidemiology, Health Promotion and Healthcare Innovation) were organized. All focus groups were recorded using a voice recorder. The children’s focus groups were held at their school or after-school care facility; the focus group with the researchers took place at the VU university. During the focus groups the children and researchers started with testing the MyDailyMoves measurement tool by recording their activities on a timeline on the website. A subsample of the children (*n* = 1–2 per focus group) were asked to ‘think aloud’ while filling in the measurement tool, recording all their thoughts and comments using a voice recorder. After examining the MyDailyMoves measurement tool the children and researchers discussed the relevance of all items/questions, the understandability, and the comprehensiveness of the tool using a topic guide based on the sequence of the items/questions in the tool. To analyze the data, recordings of all focus groups were transcribed. Next, the transcriptions were coded by two researchers. The sequence of the webpages, including the questions in the measurement tool, was used as a coding scheme, i.e. for each web-page/question within MyDailyMoves, the mentioned adaptations regarding comprehensiveness and understandability were coded. Next, the adaptations mentioned by the different groups of children and researchers were combined/summarized per webpage/question of MyDailyMoves. Lastly, two researchers discussed the summaries of the mentioned adaptations and decided on the final adaptations to the MyDailyMoves measurement tool. In case of discrepancies between researchers’ and children’s perceptions, children’s opinions were prioritized.

## Results

### Participants and schools (step 1, 2 and 4)

Forty children (40% boys) aged 10.3 ± 1.0 from two more rural-located schools (one medium and one high SES) and two urban-located schools (one medium and one high SES) participated in the concept mapping study (response rate 39%). Twenty-four children (50% boys) aged 9.9 ± 1.1 from three urban-located schools, of which two were located in the city center (one low- and one high-SES) and one in the suburbs (medium-SES), participated in the photovoice study (response rate 21%). Due to illness, two children missed one photovoice meeting, and five children dropped out: one child after the first session, two children after the second session, and two after the third session. Reasons for drop-out were the following: a child claiming that all his activities were already photographed (*n* = 1), parental concerns about their child working individually on the project (*n* = 1), and children losing interest after the second or third session (*n* = 3). In the content validity study, 22 children (36% boys) aged 10.1 ± 0.9 (response rate 56%) and seven researchers (response rate 100%) participated. The children were recruited from three urban located schools/after school-care facilities of which all were located in the suburbs (two low- and one high-SES).

### Step 1 - Concept mapping

The children collectively indicated that they engaged in 93 to 98 different physical activities. Table 1 shows the activity categories obtained from the concept maps of the schools: 1) playing (outside), 2) sport, 3) hobbies, 4) chores, 5) personal care, 6) walking/transport, 7) trips/getaways. Moreover, Table 1 shows for each activity the frequency and intensity averages and ranges across all schools. Activity frequency ratings ranged from 1.7 (trips/getaways) to 4.3 (personal care). Activity intensity ratings ranged from 1.3 (personal care) to 2.8 (sport). The concept maps of school 1 to 4 as well as the average frequency and intensity ratings of the individual activities and activity categories for each school can be found in Additional files 1 and 2, respectively.

**Table 1:** Activity categories with average frequency and intensity ratings, and specific activities and locations

**Activity category: Playing (outside)** Frequency average^a^ (range): 2.1 (1.0 to 4.8) Intensity average^a^ (range): 2.0 (1.1 to 3.6)	
**Specific activities:** Doing a cartwheel, climbing (climbing rack), inline skating, fighting, playing on the swing, dancing, playing outside, playing in the school gardens, playing soccer, playing in the playground, treasure hunt, riding Heelys outdoors, obstacle run, tree climbing, hanging out, running and jumping on the trampoline, jumping on the trampoline, knock and run/ring and run, sliding down the slide, climbing, playing tag, playing hide and seek, running, building huts, playing the floor is lava, playing badminton, playing inside (jumping/turning), jumping in puddles, dabbing, jumping, fighting/horsing around, sledding, building a snowman, throwing snowballs, having a snowball fight, hanging out on the seesaw, playing on the spring rider, walking on a frozen pond, ice skating, skateboarding, bottle flipping, playing on the ice, pillow fighting	**locations:** School (outside), school (inside), at someone else’s home (outside), at home (inside), at home (outside), in the neighborhood (outside), other
**Activity category: Sport** Frequency average^a^ (range): 1.8 (1.0 to 4.7) Intensity average^a^ (range): 2.8 (1.0 to 4.2)	
**Specific activities:** Dancing, cycling on a sports bike, acrogym, playing tennis, doing push-ups, playing soccer, gymnastics (in school), morning fitness, swimming, playing volleyball, kung fu, karate, gymnastics (at the sports club), fitness, ice skating, rowing (on a rowing machine), having a work-out, yoga, hockey	**locations:** At someone else’s home (inside), school (inside), at the sports club, in the neighborhood (inside)
**Activity category: Hobbies** Frequency average^a^ (range): 1.9 (1.2 to 2.5) Intensity average^a^ (range): 1.7 (1.0 to 2.5)	
**Specific activities:** Cheerleading, science club, shopping, walking the dog, going to the woods, doing handicrafts, cooking, drum class, taking care of a horse, drumming, going to the school gardens/doing handicrafts	**locations:** In the neighborhood (inside), in the neighborhood (outside)
**Activity category: Chores** Frequency average^a^ (range): 2.6 (1.1 to 4.6) Intensity average^a^ (range): 1.9 (1.0 to 3.4)	
**Specific activities:** Buying food at the market, tidying up, walking the dog, bringing milk around at school, buying groceries, cooking dinner, vacuuming, watering plants, selling lottery tickets	**locations:** In the neighborhood (outside), in the neighborhood (inside), at home (inside), school (inside)
**Activity category: Personal care** Frequency average^a^ (range): 4.3 (1.7 to 5.0) Intensity average^a^ (range): 1.3 (1.0 to 1.8)	
**Specific activities:** Packing a bag, brushing my hair, brushing my teeth, tying my shoelaces, preparing food, eating, taking a shower, washing my hands	**location:** At home (inside)
**Activity category: Walking/transport** Frequency average^a^ (range): 3.7 (2.0 to 5.0) Intensity average^a^ (range): 1.8 (1.2 to 2.7)	
**Specific activities:** Cycling, walking, walking up/down the stairs, strolling, running	**locations:** School (inside), school (outside), in the neighborhood (outside)
**Activity category: Trips/getaways** Frequency average^a^ (range): 1.7 (1.0 to 2.4) Intensity average^a^ (range): 2.3 (1.1 to 3.4)	
**Specific activities:** Visiting grandmother, playing outside, going on a school trip, snowboarding, skiing, bowling, pool party, children’s birthday party, playing glow-in-the-dark golf, visiting aunt and uncle, building a snowman, pillow fight	**locations:** At someone else’s home (inside), at someone else’s home (outside), in the neighborhood (outside), in the neighborhood (inside), other

^a^Ratings ranging from 1 to 5, the higher the score the higher the frequency or intensity

### Step 2 - Photovoice

The number of photographs ranged between 197 and 434 (week and weekend days combined) between schools. Photographs and written down activities of the different schools represented between 27 and 96 unique combinations of activities, locations, and moments on weekdays, and between 20 and 50 unique combinations on weekend days. In Table 1 all activities and locations are sorted using the identified activity categories.

### Step 3 - Development of MyDailyMoves

*Format of MyDailyMoves:* We built on the most promising available physical activity questionnaire(s) for youth. The most recent systematic review on the measurement properties of physical activity questionnaires for youth [20] concluded that for adolescents, a questionnaire using a segmented day structure was most valid, which recalled the previous 3 days. As no evidence for valid questionnaires for younger children was found, we chose to build on this segmented format. During the photovoice study, a segmented day structure was realized by using a timeline which included important timeframes, e.g. school breaks and the start and end of school. The photovoice study showed that the children understood and found it easy to work with the timeline. Children added relevant timeframes to the timeline, such as the start of school, school breaks, and the end of school, which facilitated the placement of the photographs of their activities in different time segments. Therefore, we chose to use a timeline structure within the measurement tool that includes the aforementioned time markers. The timeline can be personalized based on the wake-up and sleep-time of the children, which can be filled in before recording activities on the timeline. We chose to develop an online measurement instrument as the timeline can be personalized for each child, and it is easier to administer (both for children and researchers) when compared to a paper-based questionnaire. Furthermore, according to the previously-mentioned review, the most valid questionnaire in adolescents measured the previous 3 days. As recalling activities can be difficult, especially for children [18, 39], MyDailyMoves measures the previous day (the shortest recall period possible). When children use the tool on a Monday a Saturday or Sunday is randomly selected. This can be repeated during several days to obtain more data.

*Content of MyDailyMoves:* MyDailyMoves records children’s activities in 11 categories: 1) playing inside, 2) playing outside, 3) hobbies, 4) chores, 5) sports, 6) transport (active and passive), 7) schoolwork, 8) personal care, 9) screen time, 10) eating, and 11) others. The first seven categories were based on the results of the concept mapping and photovoice study. Two more sedentary categories (i.e. screen time and schoolwork) were added based on evidence from the systematic review on the measurement properties of sedentary behavior questionnaires for children [21], and an ‘others’ category was added. The category ‘eating’ was added based on the content validity study. The trips/getaways category found in the concept mapping study was not included as children indicated that the frequency of these activities is low (i.e. less than 1–2 days per week), yet children can record these activities using the ‘other’ option. The concept mapping playing (outside) activity category was split up to be able to distinguish between playing inside and outside. For the ‘sports’ category, MyDailyMoves also records the specific sport that the child engaged in, as the intensity of the activity varies considerably across sports [40]. A previous review stated that at least screen time, school/study time, passive transport, and quiet play/hobbies/social activities should be included to measure all sedentary behavior (sub)constructs [21]. MyDailyMoves already included the categories transport and playing inside and hobbies, yet based on this review, the more sedentary categories ‘screen time’ and ‘schoolwork’ were added. As the concept mapping study demonstrated that children categorize some of the activities differently, e.g. walking the dog was categorized as walking/transport and as playing (outside) or a hobby, children can decide for themselves which category they perceive as most appropriate.

For each main activity that the child recorded, MyDailyMoves records the location: 1) home (inside), 2) home (outside), 3) school (inside), 4) school (outside), 5) in the neighborhood (inside), 6) in the neighborhood (outside), 7) at the sports club, 8) at someone else’s home (inside), 9) at someone else’s home (outside), and 10) other. The first nine categories were based on the results of the photovoice study; additionally, the ‘other’ option was added.

MyDailyMoves also records the perceived exertion of each main activity. Perceived exertion is rated on an 11-point semantic-scale: 0 indicates ‘not at all sweaty, tired and/or breathless’; and 10 indicates ‘so sweaty, tired and/or breathless, I can’t go anymore’. Six illustrations are placed alongside the perceived exertion scale to clarify the scores, with one being of a child in a sedentary position on the left (0) and a running child whose heart is beating fast and who is sweating on the right (10); the illustrations in between increase in intensity. The word ‘tired’ was added to the explanation, and four of the six illustrations were added based on the content validity study.

Additionally, MyDailyMoves records questions regarding personal characteristics (i.e. age and sex) before filling in the timeline and questions regarding sports club membership, whether the day recorded on the timeline was an ‘ordinary’ day (e.g. considering illness) and questions about their sleep (i.e. sleep duration, efficiency, timing, quality, and daytime sleepiness) after filling in the timeline. One or two questions were included per sleep domain: 1) “What time did you go to sleep last night?” and “What time did you wake up this morning?” for sleep duration; 2) “When I tried to fall asleep last night, … A) I fell asleep immediately, B) I stayed awake for a little while, C) It took me a long time to fall asleep” and “How many times did you wake up last night? A) none, B) one time, C) two times, D) three times or more” for sleep efficiency; 4) “How did you sleep last night?” on a 5-point Likert-scale ranging from very bad to very good for sleep quality; and 5) “How often did you feel sleepy yesterday during the day?” on a 5-point Likert scale ranging from ‘not at all’ to ‘very often’ for daytime sleepiness. Lastly, based on the content validity study in the final version, a comments box was added in which children could give additional information that they wanted to add.

Importantly, we have put a lot of emphasis on making MyDailyMoves appealing and understandable for children by designing clear and appealing illustrations of the activity categories, locations, perceived exertion scale and the instructions for using the tool. For an example of a filled in timeline, an explanation (using MyDailyMoves images) of how activities can be added to the timeline and a preview of the MyDailyMoves format, see Additional file 3.

*Output of MyDailyMoves:* the output of MyDailyMoves includes an intensity rating for each individual activity and/or location that is based on the rating on the perceived exertion scale and Metabolic Equivalent values (METs) from the Compendium of Energy Expenditure (EE) for Youth which is developed to estimate intensity levels of questionnaire-based physical activities [40]. Subsequently, time spent sleeping and in sedentary behavior, light, moderate, vigorous and moderate-to-vigorous intensity physical activity will be calculated either for each specific activity category and/or location or time spent during a specific time period, e.g. per day. We will use two different methods to calculate time estimates of sedentary behavior (SB), light (LPA), moderate (MPA), and vigorous intensity physical activity (VPA): 1) existing cut-points on the 11-point perceived exertion scale to classify scores into four intensity categories, i.e. SB (< 1), LPA (1–2), MPA (3–4), and VPA (> 5) [41]; 2) METs from the Compendium of Energy Expenditure for Youth [40]. Subsequently, the MET-value cut-points will be used to classify the MET-values into light (1,5- < 3 METs), moderate (3- < 6 METs), and vigorous intensity physical activity (≥6 METs). In addition, the output shows the recordings of children’s personal characteristics and the different sleep domains.

### Step 4 - Content validity of MyDailyMoves and finalizing the tool

#### Usability

The children thought it was fun to use MyDailyMoves to record their activities during the day: *‘Fantastic!’ (child; group 1*), ‘*It looked very professional!*’ *(child; group 1), ‘I liked it, also because we were allowed to do it on a laptop or iPad.’ (child; group 2).* However, there were some issues regarding the usability of the tool that needed to be improved according to the children. Improvements mainly concerned making it easier to fill in MyDailyMoves, e.g. being able to add an activity to the timeline in multiple ways, using a search option to find the right sport, and being able to save what you have already filled in when you need to go back to a previous page: *‘Well if, for example, you made a mistake and went back, then the activities you had filled in on the timeline don’t get saved, so, what I’d already filled in was gone and I had to do it all over again because I filled it in wrong again, and again it wasn’t saved.’ (child; group 3*). In addition, requiring all questions to be filled in before entering the next page/question, using more illustrations (on each page) to make MyDailyMoves more attractive, and being able to correct mistakes (e.g. using a back button*)* were mentioned: *‘At one point, after I had clicked on something, and then I thought it was wrong, I wanted to delete it, but it became green and I couldn’t change it anymore.*’ *(child; group 1).* The researchers agreed with the children that the tool was attractive but quite complicated: *‘It looks attractive, but I found it quite complicated.’ (researcher).*

#### Comprehensiveness

According to both children and researchers a number of specific sports were missing in the sports category: *‘Were there any sports missing from the list? Sports that you do… (interviewer; group 2)’, ‘dodgeball, softball!’ (child; group 2). ‘I received a list of sports that children play, there are a lot of sports on my list that are missing from yours. Those are new sports that have appeared in the last 10 years.’ (researcher).* Therefore, missing sports were added (e.g. acrobatics, netball, floorball) in the second version of MyDailyMoves. Furthermore, children mentioned that for some questions answering categories were missing, e.g.: *‘Can you also add one to do with eating, or something like that?’ (child; group 3),’ Well, for the gender, you can also, for example, if you are a child, you can very quickly find out what your gender is, so maybe you can add an ‘other’ option.’ (child; group 3).* Therefore, the activity category ‘eating’ and the gender option ‘other’ were added. In addition, children indicated that the perceived exertion scale missed the ability to fill in decimals, (e.g. 0.5 – 1.5 – 2.5, etc.), which were therefore added to the second version of MyDailyMoves. Lastly, children and researchers mentioned the importance of being able to fill in additional information in addition to the standard questions: *‘Were there any other things we could ask as additional questions’ (interviewer; group 1), ‘Maybe something about school, what do you think about school, do you like school?’ (child; group 1). ‘There’s no room for a child to indicate that he/she has asthma, for example. Maybe we can add a general question like, do you want to share anything else, then a child suffering from asthma can maybe share that he/she was out of breath a lot of the time, but that it was normal for him/her.’ (researcher).* Since the children mentioned a wide variety of potential additional questions not directly relevant for measuring physical activity, sedentary behavior or sleep, a general comments box was included in the second version of MyDailyMoves where children can share anything they want.

#### Understandability

According to both children and researchers the amount of text used to explain the tool was too much and too complicated for the children: *‘I didn’t understand any of that.’ (child; group 1), ‘I think it’s really stupid to have to read a long explanation. Then it’s going to be super boring.’ (child; group 2).* Therefore, both children and researchers recommended adding an instructional video. Moreover, some of the questions or answering options should be renamed to match the children’s vocabulary. For example, the word ‘screen time’ was too difficult; instead, examples (e.g. watching TV, gaming) should be used: ‘*So, suppose we add examples to the ‘screen time’ category, such as mobile phones, watching TV, gaming. And then ‘screen time’ in brackets, to show that all examples belong to screen time. Is it clearer that way?’ (interviewer; group 3), ‘Yes! (multiple voices)’ (children; group 3).* In addition, children mentioned that the word ‘tired’ should be added to the perceived exertion scale and that more illustrations should be added alongside the scale, displaying a child that increases his/her intensity. *‘Yeah, what I said before, […], like small beads of sweat here, no beads of sweat there, small beads of sweat here, and then bigger beads of sweat there until there are lots of beads of sweat.’ (child; group 3).* Furthermore, children sometimes forgot for which day they were supposed to fill in the timeline; therefore, the recall day should always be shown above the timeline. Lastly, some of the illustrations had to be adapted to improve the understandability, e.g. a bus should be added to the illustrations displaying passive transport. All these improvements were adapted in the second version of MyDailyMoves.

#### Relevance of items

The children did not indicate any irrelevant items within the tool. Some of the researchers mentioned that the number of locations included in the tool was rather extensive. However, as the children did not mention this as a point for improvement, the number of locations was not restricted in the second version of MyDailyMoves.

## Discussion

The aim of this study was to co-create a 24-h movement behavior tool jointly with 9–12-year-old children that measures physical activity, sedentary behavior, and sleep. The developed tool, MyDailyMoves, is an online measurement tool recalling the previous day using a timeline format and including timeframes (e.g. start of school, end of school, school breaks). Children can record their activities, the intensity, and locations of their activities on the timeline. Furthermore, children can report their sleep duration, efficiency, timing, quality, and daytime sleepiness. The focus groups indicated that MyDailyMoves is a tool that uses a child-friendly format and language and includes all activities that children engage in within a 24-h timeframe.

Compared to existing physical activity, sedentary behavior, and sleep questionnaires in children, MyDailyMoves adds novel features. First, children record all their activities separately on a timeline within MyDailyMoves, thereby preventing that children have to recall all activities of a specific intensity (i.e. sedentary, light, moderate or vigorous) and add up the total time spent in that intensity. Second, MyDailyMoves uses a segmented day structure with a start- and an end-time for its timeline, resulting in less room for over- or underestimation when compared to most original-paper-based-questionnaires. Third, MyDailyMoves is an online format which gives the opportunity to personalize the timeline to the specific child, e.g. wake-up and sleep-time, and school-times. Fourth, a minimum number of sleep-related questions is included while taking into account children’s attention span and nevertheless covering all sleep domains based on a pre-defined concept of sleep [38, 42]. In contrast, most paper-based sedentary behavior or physical activity questionnaires include questions regarding the duration and/or frequency of engaging in different physical- or sedentary activities such as asking children to estimate the total time (in minutes/hours) they spent on sport or watching TV on the previous day. Such questions increase the chance of over- and/or underestimation as children can fill in as much or as little time as they think. Furthermore, only two multidimensional sleep self-reports for children exist according to a systematic review on sleep questionnaires [43]; both include an extensive list of questions, and only one includes all sleep domains: the Sleep Habits Survey (SHS). The SHS asks children to think about their sleep in general and how they ‘usually’ sleep [44]; this asks children to recall multiple nights and summarize their sleep experiences in one single answer, which might be a difficult task for children. Moreover, the SHS includes 63 items, which also requires a long attention span.

Two measurement instruments using a similar segmented format exist: the 3-Day Physical Activity Record (3DPARecord) (paper-based) [45] and the MARCA (online instrument) [46]. The 3DPARecord divides the day in 96 15-min periods, where for each time segment the children fill in their energy expenditure ranging from 1 (sleep) to 9 (vigorous physical activity and sport). Unfortunately, this questionnaire lacks information on the specific activity that the child is doing. Furthermore, only the sleep domain ‘duration’ was included. The MARCA is a previous day use-of-time instrument that asks the children to set their own time frames (e.g. school breaks) and record their activities within each timeframe by using an activity compendium including over 200 activities. However, the activities included were not based on children’s perception of physical activity. Moreover, choosing a specific activity from over 200 requires a long attention span. Lastly, the MARCA does not include sleep.

### Strengths & limitations

A major strength of this study is the involvement of the children in both the development and the content validity assessment phase, as children are the experts of their own behavior and know best which activities are relevant to capture their 24-h movement behaviors. Another strength of this study is the content validity assessment with both researchers and children, which confirmed that no relevant activity items were missing and that all included items were relevant. Furthermore, we achieved triangulation by using the concept mapping method to examine children’s perceptions of physical activities in combination with the photovoice method to assess children’s actual activities and the focus groups to examine content validity. In addition, we followed the COSMIN protocol for content validity which further strengthens our study [23]. Moreover, within each school/after-school care facility data were collected until saturation was achieved, thereby supporting the representativeness of our findings. Furthermore, although a small number of children participated in each phase of the study, it was nonetheless a diverse group of children selected from schools and after-school care facilities of different SES located in urban and rural areas, which further supports the representativeness. A limitation of our study is the primary focus on physical activity at the start of the study and including sedentary behavior and sleep in a later phase. Consequently, we did not obtain children’s perceptions regarding sedentary behavior and sleep, thereby possibly missing out on potential relevant sedentary activities and sleep behavior questions. However, during the focus groups children had no comments regarding missing sedentary behavior categories and mentioned that the most important sleep questions were included. Lastly, as MyDailyMoves is a Dutch tool, only Dutch children and researchers were involved in the development. Whether the tool is also suitable and comprehensible for children in other countries needs further research.

## Conclusion

Including children’s perceptions in the different phases of the development and the content validity assessment of MyDailyMoves resulted in a measurement instrument that is comprehensive, practical, easy-to-use and relevant for children. Furthermore, MyDailyMoves is the first personalized 24-h movement behavior child-report tool, including physical activity, sedentary behavior, and sleep. Assessment of the construct validity and the test-retest reliability of MyDailyMoves is the next step to conclude on the quality of MyDailyMoves.

## Supplementary information

**Additional file 1.** Concept maps. Four concept maps, one for each school class.

**Additional file 2.** Cluster compositions, and frequency and intensity ratings. Cluster compositions and average frequency and internsity ratings of the underlying ideas.

**Additional file 3.** MyDailyMoves images. An example of a filled in timeline, an explanation (using MyDailyMoves images) of how activities can be added to the timeline, and a preview of the MyDailyMoves format.

## Data Availability

All data generated or analysed during this study are included in this published article and its supplementary files.

## Abbreviations

COSMIN: COnsensus-based Standards for the selection of health Measurement INstruments
SES: Socio-economic status
METs: Metabolic Equivalent values
SB: Sedentary behavior
LPA: Light physical activity
MPA: Moderate physical activity
VPA: Vigorous physical activity
SHS: Sleep Habits Survey
3DPARecord: 3-Day Physical Activity Record
