# Supplementary material for: Co-creating a 24-hour movement behavior tool together with 9–12-year-old children using mixed-methods: MyDailyMoves
Source: Int J Behav Nutr Phys Act. 2020 May 14;17:63. doi: 10.1186/s12966-020-00965-0 (PMC7226934; doi:10.1186/s12966-020-00965-0)
Supplement: Supplementary file 1 — Additional file 1. Concept maps. Four concept maps, one for each school class. [file 12966_2020_965_MOESM1_ESM.docx]

Additional file 1: Concept maps

**
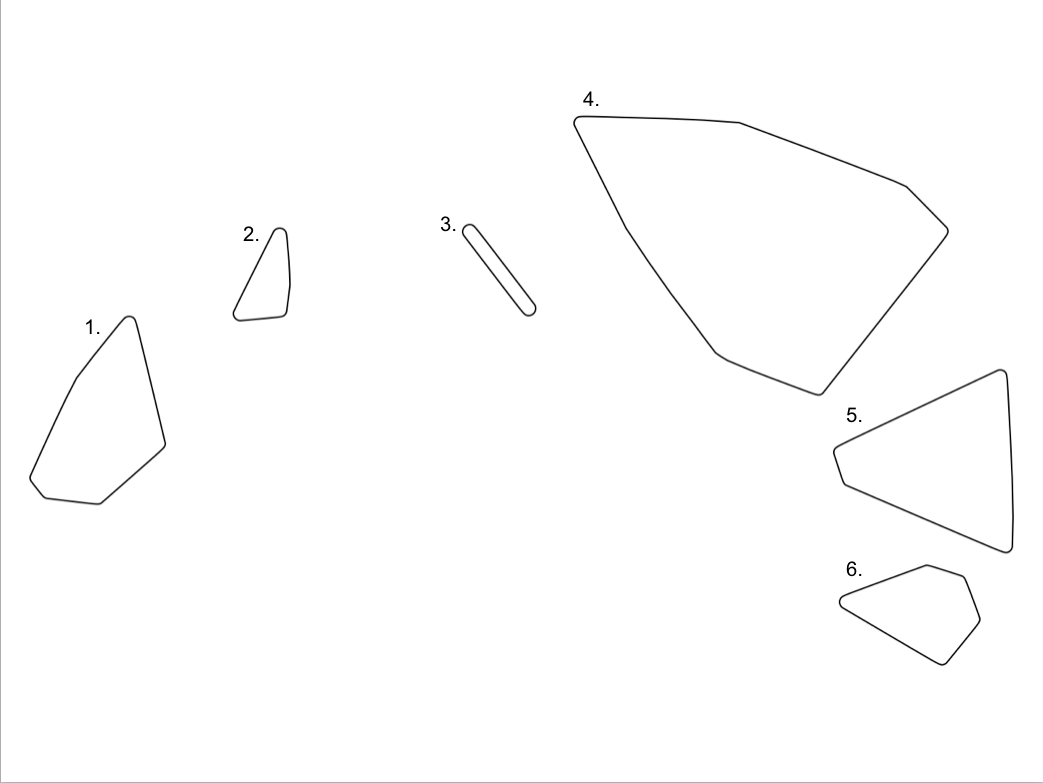
**

**Figure 1.** Concept map school 1

Note that clusters closer together on the map have a stronger relation than clusters further away from each other.

Cluster 1: Sports; Cluster 2: Hobbies; Cluster 3: Trips/getaways; Cluster 4: Playing (outside); Cluster 5: Walking/transport; Cluster 6: Chores and personal care.

**
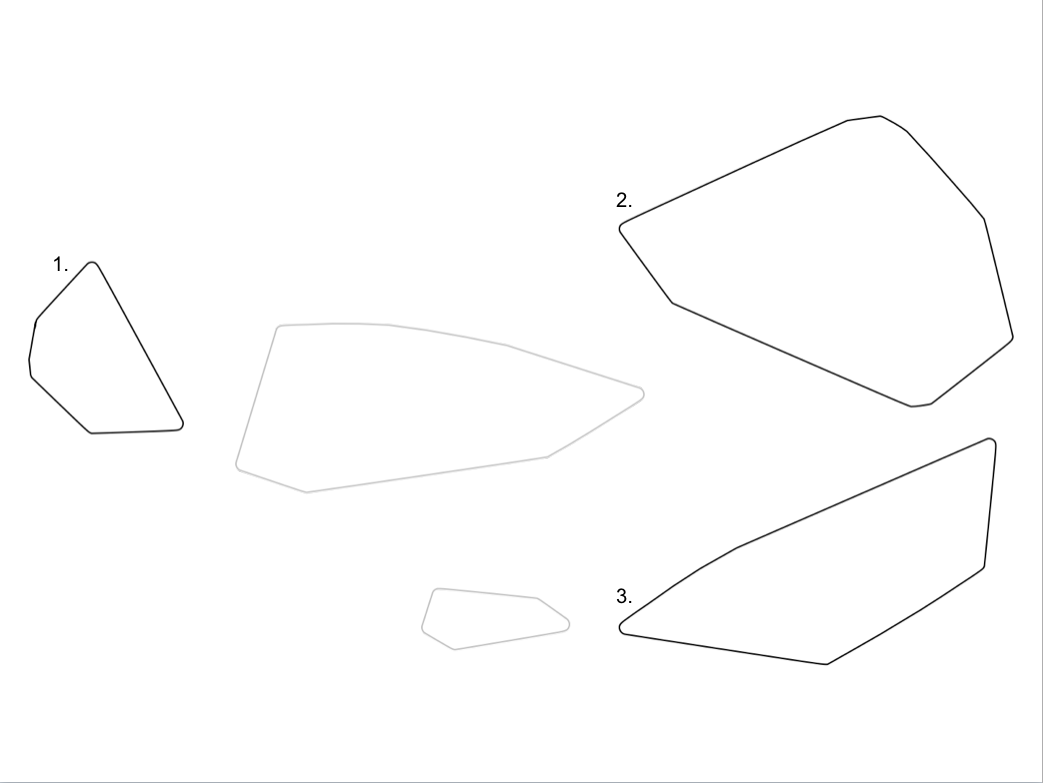
**

**Figure 2.** Concept map school 2

Note that clusters closer together on the map have a stronger relation than clusters further away from each other.

Cluster 1: Sports; Cluster 2: Hobbies, chores, personal care and walking/transport; Cluster 3: Playing outside and trips/getaways

Note that the light grey clusters has disappeared as a result of reallocation of statements.


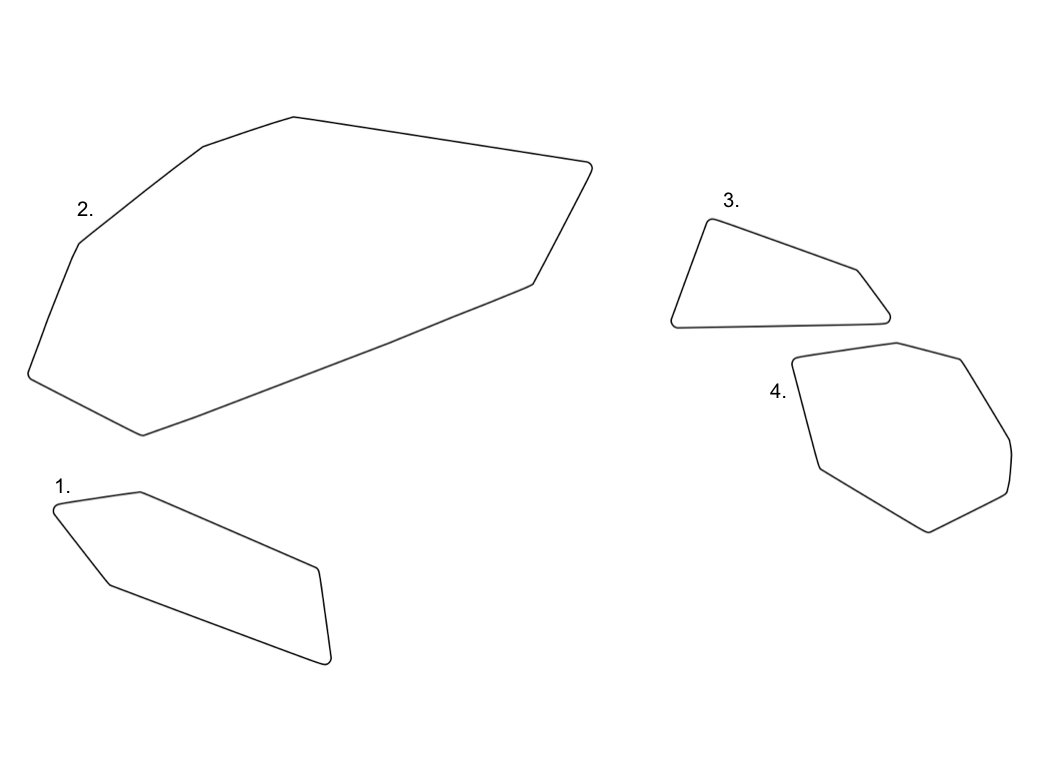


**Figure 3.** Concept map school 3

Note that clusters closer together on the map have a stronger relation than clusters further away from each other.

Cluster 1: Personal care and hobbies; Cluster 2: Chores, playing (outside) and trips/getaways; Cluster 3: Playing (outside); Cluster 4: Sports and walking/transport

**
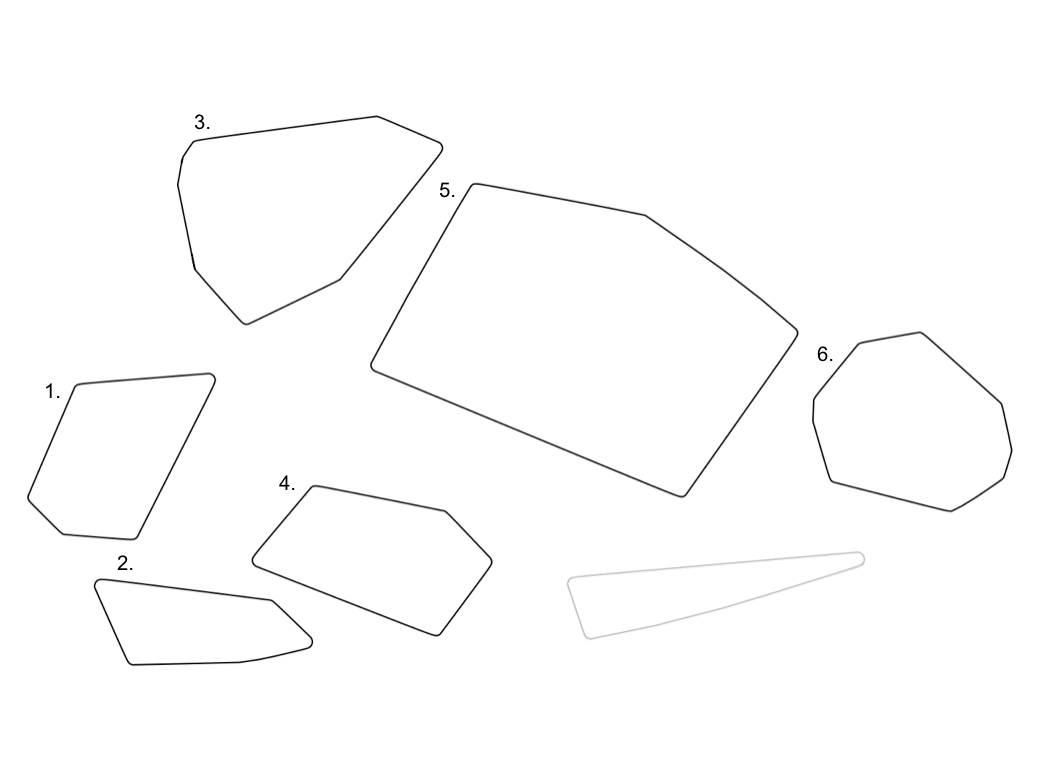
**

**Figure 4.** Concept map school 4

Note that clusters closer together on the map have a stronger relation than clusters further away from each other.

Cluster 1: Playing (outside); Cluster 2: Personal care and chores; Cluster 3: Playing (outside); Cluster 4: Transport/walking and hobbies; Cluster 5: Trips/getaways; Cluster 6: Sports

Note that the light grey cluster has disappeared as a result of reallocation of statements.
