# Supplementary material for: Co-creating a 24-hour movement behavior tool together with 9–12-year-old children using mixed-methods: MyDailyMoves
Source: Int J Behav Nutr Phys Act. 2020 May 14;17:63. doi: 10.1186/s12966-020-00965-0 (PMC7226934; doi:10.1186/s12966-020-00965-0)
Supplement: Supplementary file 2 — Additional file 2. Cluster compositions, and frequency and intensity ratings. Cluster compositions and average frequency and internsity ratings of the underlying ideas. [file 12966_2020_965_MOESM2_ESM.docx]

## Additional file 2: Cluster compositions, and frequency and intensity ratings

Table 1. Clusters, underlying activities, frequency and intensity ratings of school 1

|  | Clusters and statements | Frequency | Intensity |
| --- | --- | --- | --- |
| 1. | Sports  1. (Indoor) soccer  2. Ice hockey  3. Physical education  5. Gymnastics 6. Tennis 9. Ice skating  11. Climbing  14. Cycling 16. Hurdling  19. Swimming  23. Dancing  25. Running 26. Field hockey  32. Kenpo karate 38. Mountaineering  43. Basketball 44. Fighting  46. Kickboxing  50. Skiing  59. Sailing  64. Mat soccer (to score on the mat of the other team) 73. Dodgeball  75. Ballet  76. Skating  80. Rugby  81. 'Apenkooien' (Dutch tagging game typically played during physical education classes; is characterized by the hindrances set up in the gym)  83. Korfball  91. Surfing  93. Volleyball  94. Playing golf  95. Relay race | 1.90  1.20  2.60  1.30  1.30  1.80  1.80  3.20  1.70  2.20  1.50  4.30  1.30  1.30  1.40  1.90  1.40  1.70  1.30  1.20  2.00  2.30  1.20  1.60  1.00  1.50  1.70  1.40  1.30  1.30  1.70 | 3.10  3.50  2.70  2.20  3.20  3.20  2.60  2.40  3.10  2.50  2.50  3.10  3.30  3.10  3.70  3.40  3.20  4.20  2.90  2.60  3.10  3.10  2.00  2.00  3.90  3.00  3.00  3.30  3.50  2.20  2.90 |
| 2. | Hobbies  67. Darts  96. Scouting  29. Cooking^a^ | 2.00  1.40  1.70 | 1.90  2.20  2.20 |
| 3. | Trips/getaways  20. Cartwheel  57. Bowling  71. Camping^a^  65. (Scuba) diving^a^  92. Waterskiing^a^ | 1.60  1.90  1.60  1.90  1.40 | 1.50  2.40  2.00  2.30  3.40 |
| 4. | Playing (outside)  4. Gaming actively (e.g. Wii™)^a^  10. Handstand^a^  12. Crafting (e.g. painting)  22. Throwing leaves  28. Acting  31. Searching for chestnuts in the woods^a^  33. Playing outside  34. Snowball or stick fight  35. Crawling  36. Twister™  37. Hoola-hooping 39. Playing catch  40. Playing tag  41. Playing (alone or with friends)  42. Sleighing 45. Cheering (when I score)^a^  47. Playing shuffleboard  48. Playing pool  49. Making a fire  51. Walking on tin cans  54. Lighting fireworks 55. Jumping  56. Hopscotching  58. Rollerblading^a^  63. Building a snowman 66. Airhockey^a^  68. Hide-and-seek  69. Picking flowers^a^  70. Riding a kick scooter  74. Climbing a tree  77. Ding-dong ditch  78. Fishing  79. Building a sand castle^a^ 82. Playing with Lego®^a^  84. Skipping ropes 86. Tussling^a^  87. Building a hut or tree house  88. Sack-race^a^ | 2.50  1.80  2.70  1.70  1.40  1.50  4.20  2.30  1.40  1.60  1.30  2.40  2.30  3.90  1.80  1.90  1.50  1.60  1.70  1.10  1.90  2.50  1.50  2.00  1.60  1.50  2.00  1.70  1.70  1.80  1.60  1.70  1.50  2.10  1.40  1.60  1.90  1.30 | 1.50  1.70  1.40  1.90  1.90  2.50  2.50  2.80  1.80  2.40  1.90  2.10  3.20  2.10  2.10  1.40  1.60  1.70  1.70  1.80  1.80  2.10  1.90  2.70  1.80  1.90  2.10  1.20  2.00  2.60  2.40  1.70  2.00  1.50  2.60  3.60  3.60  2.80 |
| 5. | Walking/transport  13. Walking  21. Strolling  24. Walking the dog  52. Actively traveling somewhere^a^ | 5.00  4.10  2.10  2.50 | 1.90  2.30  1.70  2.20 |
| 6. | Chores and personal care  7. Tooth brushing  8. Eating (e.g. while walking)  15. Stretching  17. Walking down the stairs  18. (Un)dressing  27. Going to or getting out of bed  30. Tidying up  53. Packing bag for school  60. Class/school housekeeping chores^a^  61. Shopping/running errands^a^  62. Taking care of animals (e.g. feeding and petting)^a^  72. Gardening^a^  85. Scraping ice of a car windshield  89. Setting the table  90. Washing the car  97. Cleaning  98. Lawn mowing | 5.00  5.00  4.60  4.90  5.00  5.00  2.80  4.60  2.20  2.70  3.00  1.60  1.60  3.20  1.70  2.40  1.30 | 1.00  1.10  1.10  1.20  1.20  1.80  2.60  1.20  1.70  1.90  1.80  2.30  1.90  1.40  2.40  2.20  2.10 |

^a^ Indicates a statement is reallocated by researchers

Table 2. Clusters, underlying activities, frequency and intensity ratings of school 2

|  | Clusters and statements | Frequency | Intensity |
| --- | --- | --- | --- |
| 1. | Sports  1.Sailing 4. Dancing 6. Skiing 8. Gymnastics 15. Ice skating 16. Racquetball 21. Kickboxing 24. Swimming 26. Badminton 32. Soccer 33. Tennis 37. Horseback riding 42. Boxing 43. Road bicycle riding 52. Baseball 53. Basketball 56. Cheerleading 61. Handball 64. Winter sports 66. Volleyball 70. Field hockey 75. Ball games 81. Ping pong 85. Rugby 91. Ballet  5. Morning workout (crunches and push-ups)^a^ 7. Goalkeeper training^a^ 14. Jogging^a^ 17. Cycling^a^ 18. Practicing the cross split and the split^a^ 23. Training at the gym (fitness)^a^ 30. Physical education^a^ 55. Practicing the handstand^a^ 68. Keepie uppie/kick-ups^a^ | 1.80  2.90  1.70  1.70  1.90  1.80  1.50  2.30  1.40  2.60  2.30  1.30  1.70  1.80  1.50  1.80  1.90  1.30  1.70  1.60  1.60  2.60  2.10  1.30  1.10  2.10  1.30  2.50  4.40  1.70  1.70  2.20  2.00  2.00 | 2.44^b^  3.00  2.90  3.30  2.80  3.10  3.70  3.10  2.40  3.60  3.40  2.90  3.50  3.50  3.40  3.40  2.40  2.90  3.80  3.20  3.10  3.40  2.50  3.50  3.30  3.50  3.60  2.90  2.20  1.80  4.10  2.80  1.90  2.10 |
| 2. | Hobbies, chores, personal care, and walking/transport  2. Walking the dog^a^  9. Walking 12. Acting  29. Playing the violin  45. Baking  69. Performing magic tricks  73. Playing the piano 76. Playing the drums 79. Playing the guitar 86. Cooking  3. Shopping^a^  34. Bird watching^a^ 20. Taking a shower  27. Tooth brushing  31. Brushing your hair  38. Getting dressed  46. Eating (e.g. while walking)  72. Dressing up  25. Babysitting small children  28. Packing a suitcase  41. Cleaning up my room  47. Cleaning  51. Doing odd jobs  63. Gardening  67. Running errands  87. Sweeping | 2.10  4.50  2.40  1.90  1.80  1.90  2.20  1.70  1.90  2.50  2.30  1.20  4.80  5.00  3.50  4.90  1.90  1.70  2.10  2.00  3.20  2.00  1.90  1.50  2.80  1.90 | 1.50  1.50  1.60  1.70  1.70  1.40  1.80  2.10  1.70  1.70  2.30  2.00  1.00  1.00  1.10  1.10  1.50  1.50  2.40  1.50  2.60  2.50  3.40  2.90  2.10  1.80 |
| 3. | Playing (outside) and trips/getaways 10. Playing outside 11. Swinging (on a swing) 13. Running away  35. Climbing 39. Building a hut  40. Bowling^a^ 48. Having a laughter attack 50. Water fight 54. Skipping ropes 60. Longboarding 65. Juggling  71. Building (K'NEX®) 77. Playing tag 78. Hide-and-seek  82. 'Blikje trap' (a specific form of hide-and-seek involving kicking away a ball which the seeker has to get before he/she can start seeking)  83. Playing 93. Throwing  22. Jumping on the trampoline^a^ 57. Riding a waveboard^a^ 58. Skateboarding^a^ 59. Rollerblading^a^ 62. Sleighing^a^  84. 'Around the table' (ping pong with a regular soccer ball and multiple players circling the ping pong table)^a^  44. Gaming^a^  74. Just Dance (Wii™)^a^  80. Twerking^a^  36. Jumping^a^  19. Travelling  49. Going to an amusement park  89. Paintballing  90. Drive go-karts^a^  92. Cartwheel^a^ | 3.60  2.20  2.50  1.80  1.60  1.90  3.70  2.00  2.10  1.70  1.30  1.60  1.90  2.10  2.00  3.80  2.50  2.40  1.60  1.50  1.70  1.60  2.00  2.50  1.90  2.50  3.00  1.90  1.90  1.80  1.70  1.90 | 1.80  1.10  3.50  2.50  2.50  2.30  1.90  2.70  2.50  2.40  1.90  1.40  2.80  2.10  2.40  2.30  1.80  2.80  2.20  2.20  2.20  2.10  2.30  1.70  3.00  1.60  2.80  2.80  2.80  2.80  3.10  2.10 |

^a^ Indicates a statement is reallocated by researchers

^b^ n=9

Table 3. Clusters, underlying activities, frequency and intensity ratings of school 3

|  | Statements and clusters | Frequency | Intensity |
| --- | --- | --- | --- |
| 1. | Personal care and hobbies  15. Playing the saxophone  26. Musical  33. Playing the piano  65. Playing drums with your hands  88. Acting  31. Getting out of bed  43. Working at school (not being able to sit still)  57. Climbing the stairs  66. (Un)dressing  72. Tooth brushing | 1.30  1.80  2.30  2.40  1.90  5.00  3.70  4.80  5.00  4.60 | 1.40  1.30  1.00  1.30  1.60  1.60  1.40  1.60  1.10  1.00 |
| 2. | Chores, playing (outside) and trips/getaways  3. Jumping  12. Being a DJ  18. TwisterTM  21. Oxboarding  23. Playing outside  24. Running (random, not as a sport)  30. Frolicking  40. Forest games  45. '"Engels tienen" (soccer game with only one goal)  61. Climbing a tree  73. Gaming  77. Seesawing  79. Riding a kangaroo ball  80. Playing shuffleboard  85. Swinging on a swing  90. "Buskruiten" (form of hide-and-seek including football)  92. Photographing  94. Playing catch (throwing and catching)  95. Rolling  96. Juggling  97. Unicycling  48. Sweeping the classroom  69. Lawn mowing  84. Pruning  68. Supping^a^  74. Waterskiing^a^  82. Rafting^a^  63. Going to a water amusement park  64. Riding a rollercoaster  86. Lasergaming  87. Paintballing | 3.70  1.80  1.60  1.50  4.80  4.00  2.30  1.70  2.40  2.60  3.40  1.60  1.60  1.80  2.40  2.40  1.80  3.10  2.70  1.40  1.00  2.10  1.20  1.10  1.20  1.00  1.20  1.80  1.80  1.70  1.30 | 1.70  1.40  1.40  1.50  2.40  3.00  1.90  2.20  1.80  2.60  1.20  1.20  1.50  1.20  1.20  1.70  1.10  1.40  1.40  1.60  1.90  1.00  1.50  1.20  2.40  2.40  2.40  1.70  1.50  2.10  2.30 |
| 3. | Playing (outside)  8. Quidditch (game played during physical education whereby foam balls and bouncy balls should be thrown against mats or through hoops)  34. Climbing  36. Jumping on the trampoline  46. Skateboarding  47. Panna King (soccer game with the aim of scoring a "panna")  50. Dodgeball  54. Rowing in a row boat  76. Skimboarding (on the beach) | 2.00  2.00  2.30  1.50  1.70  2.10  1.60  1.10 | 2.20  2.50  2.20  2.00  2.20  2.10  2.50  2.30 |
| 4. | Sports and walking/transport  1.Walking^a^  6. Cycling  2. Running (as a sport)^a^  4. Korfball  5. Field hockey  7. Swimming  9. Water polo  10. Soccer  11. Rugby  13. Baseball  14. Tennis  16. Physical education  17. Gymnastics  19. Mountain biking  20. Ice skating  22. Horseback riding  25. Badminton  27. Judo  28. Triathlon  29. Volleyball  32. Breakdance  35. Dancing  37. Dryland training (before swimming)  38. Long jumping  39. Dab (dance style)^a^  41. Charity run^a^  42. Basketball  44. Morning work-out^a^  49. Weightlifting  51. Rounders  52. Kickboxing  53. American football  55. Freerunning  56. BMX riding  58. Sailing  59. Skiing  60. Snowboarding  62. Bodyboarding  67. Kitesurfing  70. Pole vaulting  71. Softball  75. Surfing  78. Handball  83. Yoga  89. (Sea) fishing  91. Ping pong  93. Beach soccer  98. Racquetball  81. Relay race^a^ | 4.80  4.80  2.30  1.80  1.80  2.50  1.40  3.00  1.20  1.30  1.60  2.50  1.60  1.90  2.00  1.60  1.90  1.10  1.10  1.60  1.10  1.70  1.50  1.70  1.90  1.70  1.90  1.20  1.30  2.00  1.30  1.10  1.30  1.30  1.60  1.40  1.20  1.20  1.10  1.10  1.50  1.10  1.50  1.30  1.30  2.00  1.60  1.70  2.00 | 1.30  2.00  2.90  2.40  2.90  2.60  3.50  3.10  3.20  2.80  2.50  2.80  2.50  2.90  2.00  1.60  1.80  2.90  4.10  2.70  2.60  2.60  3.30  2.20  1.00  4.00  2.70  1.90  3.30  2.40  3.00  3.20  3.40  3.40  2.10  2.00  2.20  2.30  2.50  2.70  2.40  2.50  2.40  1.90  1.80  1.70  2.80  2.70  2.80 |

^a^ Indicates a statement is reallocated by researchers

Table 4. Clusters, underlying activities, frequency and intensity ratings of school 4

|  | Clusters and statements | Frequency | Intensity |
| --- | --- | --- | --- |
| 1. | Playing (outside)  21. Frolicking  48. Pillow fight  50. Jumping on a trampoline, bed or inflatable bouncer  64. TwisterTM  80. "Party & Co" (game involving impersonating something)  91. Playing shuffleboard  51. Jumping^a^ | 2.40  2.70  2.60  1.70  1.70  1.90  4.10 | 2.30  2.00  2.00  1.60  1.10  1.30  1.60 |
| 2. | Personal care and chores  29. Getting dressed  31. Climbing onto my bed (loft bed)  44. Walking the stairs  46. Standing up  37. Lifting something  38. Tidying up | 5.00  1.90  4.40  4.60  3.90  3.90 | 1.00  1.20  1.80  1.00  1.80  1.70 |
| 3. | Playing (outside)  11. Playing outside  19. Keep Away/Monkey in the Middle  26. Playing tag  28. Riding a kick scooter  30. Parkour/freerunning  32. Hide-and-seek (e.g. "blikje trap", a form of hide-and-seek  involving kicking away a ball)  35. Skating/rollerblading  55. Flying a kite  68. Snowball or water balloon fight  73. Swinging on a swing  77. Rolling in mud  78. Stratego Alive (mix between Capture the Flag and the board game Stratego)  79. "Marco Polo" (game with questions whereby you have to run back and forth)  81. Sports day  85. Quest  87. Building rafts  90. Feeding ducks  69. Throwing a ball^a^  14. "Tienen" (type of soccer game with one goal) ^a^  22. Climbing a tree^a^  53. Dodgeball^a^  71. Tumble bar^a^  84. Playing Frisbee^a^  95. Table football^a^ | 4.30  2.40  2.40  1.80  2.00  2.50  2.00  1.70  2.40  2.30  1.60  1.90  1.50  1.90  1.90  1.70  1.40  3.20  2.00  2.20  2.50  1.50  1.60  2.20 | 1.80  1.60  2.30  1.30  2.40  1.60  1.60  1.40  1.80  1.20  1.30  2.20  1.80  3.30  1.50  2.10  1.10  1.20  1.90  1.80  2.10  2.40  1.30  1.30 |
| 4. | Transport/walking and hobbies  42. Playing the guitar  45. Dancing  47. Playing the saxophone  52. Playing the piano  88. Walking the dog^a^  89. Hammering and sawing^a^  57. Strolling  60. Race walking  62. Jogging  63. Running up a hill  3. Walking^a^  2. Running (random, not as a sport)^a^ | 1.70  2.40  1.20  2.20  1.70  1.70  2.90  2.00  2.30  2.50  5.00  4.80 | 1.00  2.50  1.30  1.00  1.10  1.70  1.60  1.90  2.40  2.70  1.20  2.50 |
| 5. | Trips/getaways  5. Swimming  8. Bungee jumping  9. Windsurfing  10. Surfing  13. Sailing  17. Climbing on a climbing wall  33. Lasergaming  34. Paintballing  43. Pétanque  61. Canoeing  66. Sleighing  70. Paddle boats  72. Diving  74. Rowing  75. Bubble soccer (in a plastic ball)  76. Aquabubble (in a plastic ball on the water)  83. Polder sports "e.g. ditch jumping"  94. Fishing | 2.40  1.30  1.50  1.70  1.60  2.00  1.90  1.60  1.40  1.70  2.00  1.60  2.20  1.40  1.40  1.50  1.80  1.50 | 2.30  1.90  2.40  2.40  1.60  2.10  2.20  2.30  1.10  2.00  2.00  2.40  1.80  2.80  2.30  2.30  2.30  1.20 |
| 6. | Sports  4. Soccer  6. Cheerleading  7. Field hockey  12. Basketball  15. Skiing  18. Ping pong  20. Fencing  23. Rugby  24. Tennis  27. Gymnastics (e.g. flying rings)  36. Badminton  39. Horseback riding  40. Volleyball  41. Ice skating  49. Water polo  58. Korfball  59. Handball  67. Snowboarding  82. American football  86. Baseball  93. Frisian handball  96. Golfing  97. Ice hockey  1. Cycling^a^  25. Athletics (e.g. hurdling, discus throw, long jump, pole vault, javelin throw, shot-put, high jump, steeplechase, relay race)^a^  54. Yoga^a^  56. Running (as a sport e.g. a marathon)^a^  98. Sprinting^a^  16. Physical education^a^  92. Working out in the gym (e.g. weightlifting)^a^  65. Hockey training (to undergo or to provide it)^a^ | 3.20  1.30  3.00  2.10  2.00  2.30  1.10  1.50  2.40  1.70  2.00  1.60  1.80  2.30  1.40  1.40  1.60  1.10  1.10  1.40  1.40  1.70  1.30  4.70  1.90  1.40  2.40  3.50  3.00  1.30  2.40 | 2.40  2.30  3.30  2.50  2.60  1.50  2.20  3.00  2.70  2.50  1.90  1.50  2.50  2.50  3.40  2.20  2.30  2.40  2.90  2.60  1.90  1.70  3.00  2.30  3.30  1.50  3.70  3.50  2.40  3.00  2.60 |

^a^ Indicates a statement is reallocated by researchers
