# Supplementary material for: Co-creating a 24-hour movement behavior tool together with 9–12-year-old children using mixed-methods: MyDailyMoves
Source: Int J Behav Nutr Phys Act. 2020 May 14;17:63. doi: 10.1186/s12966-020-00965-0 (PMC7226934; doi:10.1186/s12966-020-00965-0)
Supplement: Supplementary file 3 — Additional file 3. MyDailyMoves images. An example of a filled in timeline, an explanation (using MyDailyMoves images) of how activities can be added to the timeline, and a preview of the MyDailyMoves format. [file 12966_2020_965_MOESM3_ESM.docx]

Additional file 3 – MyDailyMoves images


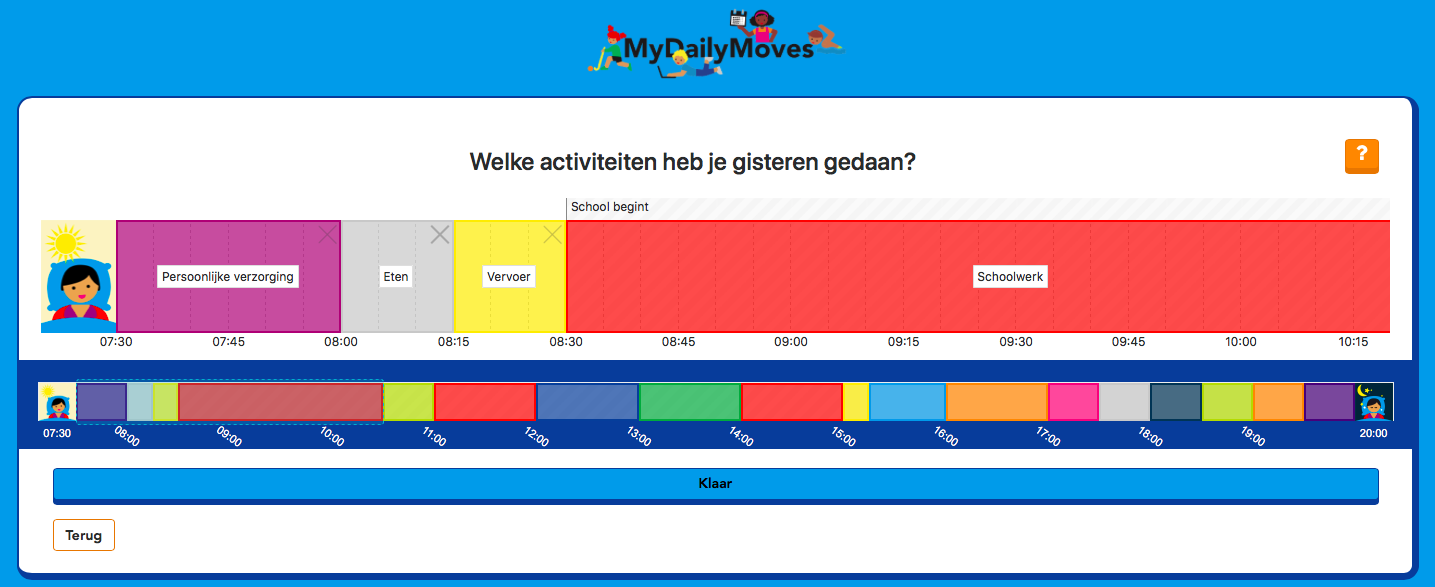


Figure 1A. Overview of the MyDailyMoves timeline

The top figure shows the beginning of a completed timeline, with each block representing a performed activity, e.g. the yellow block represents 15 minutes of transport. The small timeline at the bottom shows the complete day (i.e. from waking up until going to sleep) that is used to scroll through the timeline. The zoomed timeline at the top is used to fill in the activities.

Translation of question above the timeline: What activities did you do yesterday?


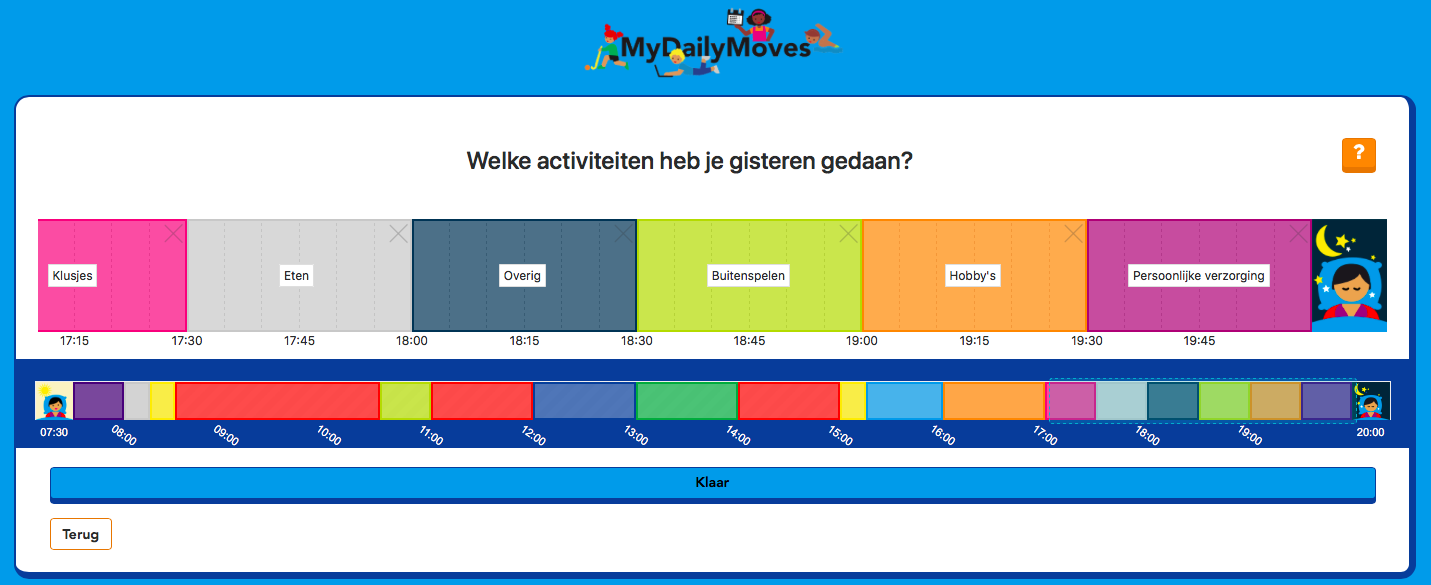


Figure 1B. Overview of the MyDailyMoves timeline

The figure shows the end of a completed timeline, with each colored block representing a performed activity, e.g. the orange block represents 30 minutes of doing hobbies.


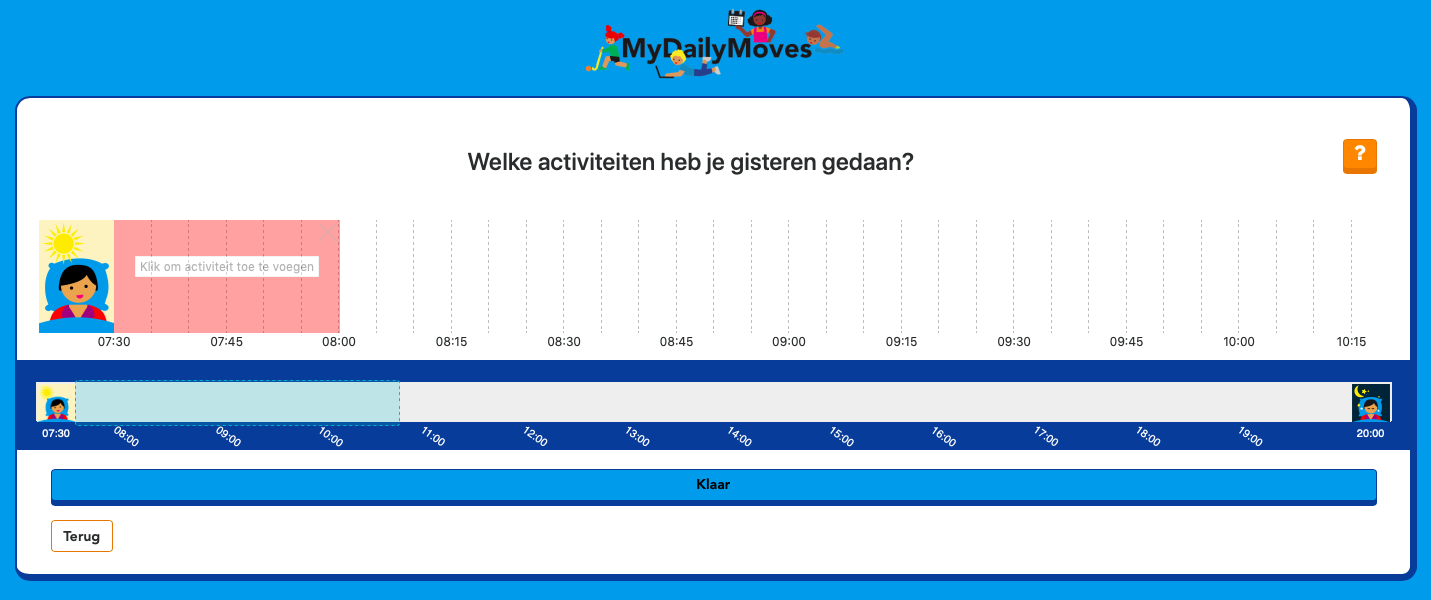


Figure 2A. Adding an activity to the timeline

An activity is added to the timeline by indicating the beginning- and end-time, as indicated by the red block. When clicking on the red block the type of activity can be selected (i.e. activity category)(Figure 2B). Subsequently, the location (Figure 2C) and a rating of the perceived exertion (Figure 2D) of the activity can be selected.


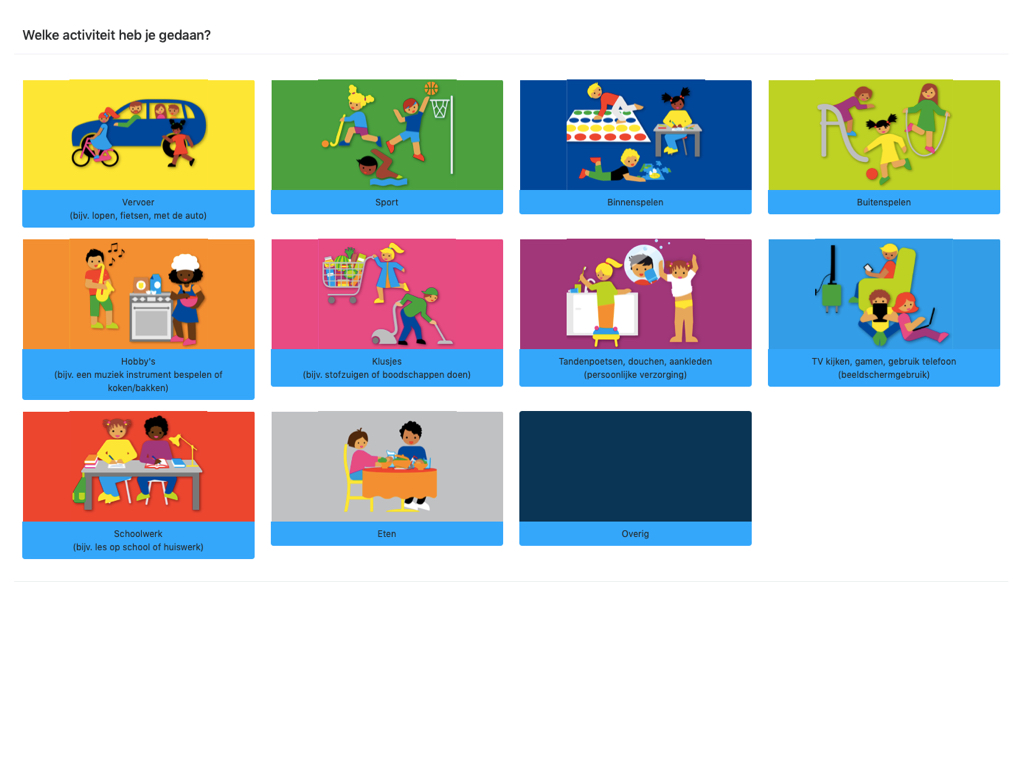
Figure 2B. Selecting the type of activity

From left to right and from top to bottom: transport; sports; playing inside; playing outside; hobbies; chores; personal care; screen time; schoolwork; eating; and others.

Translation of question above the activity categories: What activity did you do?


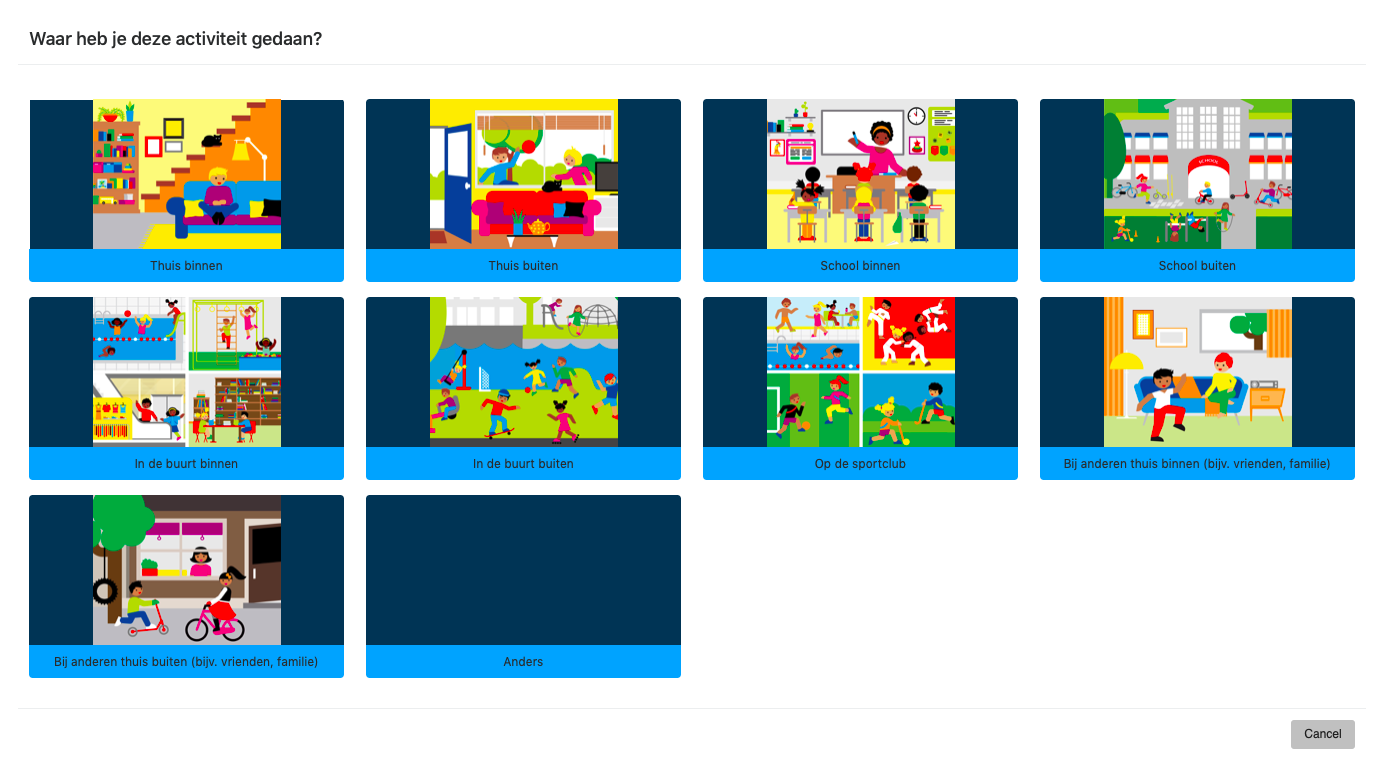
Figure 2C. Selecting the location of the activity

From left to right and from top to bottom: at home inside; at home outside; school inside; school outside; in the neighborhood inside; in the neighborhood outside; at the sports club; at someone else’s home inside; at someone else’s home outside; and other.

Translation of question above the location categories: Where did you do this activity?


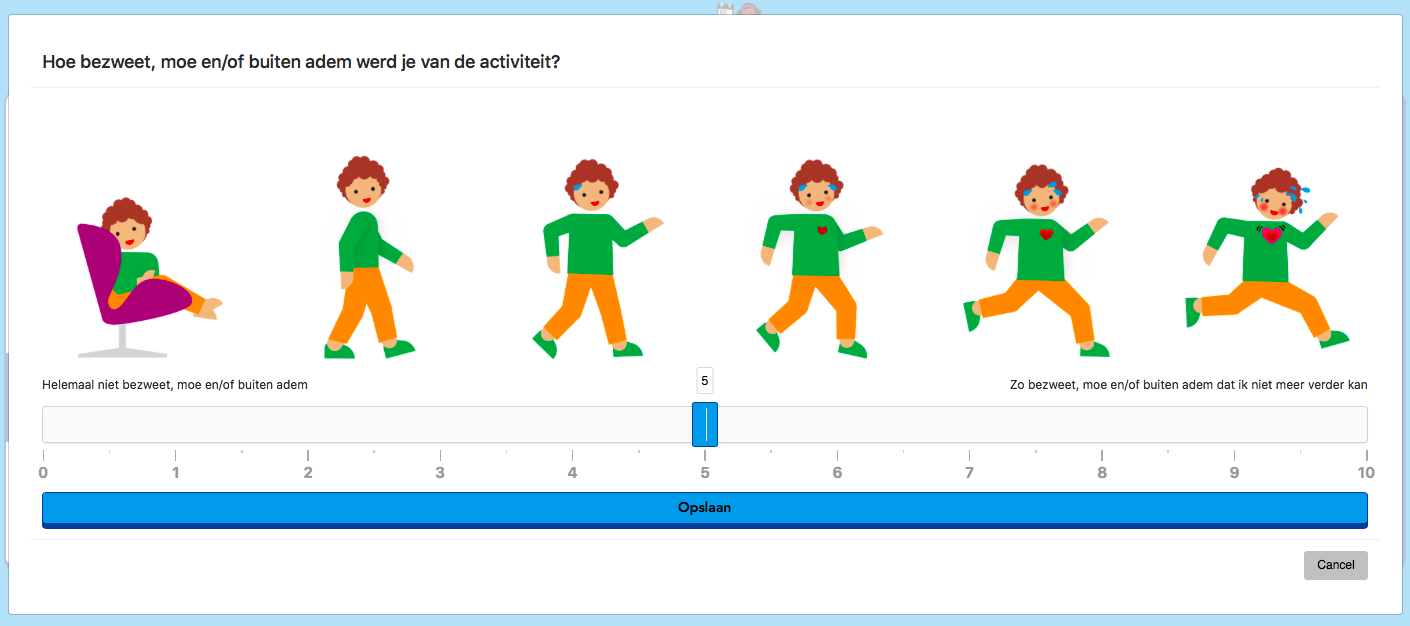


Figure 2D. Selecting the intensity of the activity

0 indicates ‘not at all sweaty, tired and/or breathless’; 10 indicates ‘so sweaty, tired and/or breathless, I can’t go anymore’.

Translation of question above the intensity scale: How sweaty, tired and/or breathless did you get from the activity?
